# *Acmella oleracea* (L) R. K. Jansen Reproductive Toxicity in Zebrafish: An* In Vivo* and* In Silico* Assessment

**DOI:** 10.1155/2019/1237301

**Published:** 2019-03-03

**Authors:** Gisele Custodio de Souza, Arlindo César Matias Pereira, Muller Duarte Viana, Adriana Maciel Ferreira, Ianna Dias Ribeiro da Silva, Monaliza Maia Rebelo de Oliveira, Wagner Luiz Ramos Barbosa, Luciane Barros Silva, Irlon Maciel Ferreira, Cleydson Breno Rodrigues dos Santos, José Carlos Tavares Carvalho

**Affiliations:** ^1^Programa de Pós-Graduação em Inovação Farmacêutica, Departamento de Ciências Biológicas e da Saúde, Colegiado de Farmácia, Universidade Federal do Amapá, Rodovia Juscelino Kubitschek, Km 02, 68902-280 Macapá, AP, Brazil; ^2^Laboratório de Pesquisa em Fármacos, Departamento de Ciências Biológicas e da Saúde, Colegiado de Farmácia, Universidade Federal do Amapá, Macapá, AP, Brazil; ^3^Programa de Pós-Graduação em Ciências Farmacêuticas, Faculdade de Farmácia, Universidade Federal do Pará, Belém, PA, Brazil; ^4^Laboratório de Modelagem e Química Computacional, Departamento de Ciências Biológicas e da Saúde, Universidade Federal do Amapá, Macapá, AP, Brazil

## Abstract

The plant species* Acmella oleracea* L. is used in the north of Brazil for the treatment of a range of illnesses, such as tuberculosis, flu, cough, and rheumatism and as an anti-inflammatory agent; besides, hydroethanolic formulations with this species are popularly used as a female aphrodisiac agent. However, currently, there are no studies performed evaluating its effect on embryonic development. Hence, this research aimed to evaluate the effects of the hydroethanolic extract of* A. oleracea* (EHFAo) on the reproductive performance (parental) and embryonic development (F1 generation) of zebrafish, at concentrations of 50, 100, and 200 *μ*g/L. Histopathology of parental gonads after 21 days of exposure to EHFAo reveals few alterations in the ovaries and testes, not impairing the reproduction; an increase of eggs deposition was observed in animals treated with EHFAo at the highest concentrations. Nevertheless, concerning the embryonic development of F1, teratogenic effects were observed including tail deformation, cardiac and yolk edema, scoliosis, and growth retardation; these alterations were more prominent in the groups born from progenitors exposed to the highest concentrations (100 and 200 *μ*g/L.); but only the occurrence of yolk and cardiac edema had a statistically significant difference when compared to the control group. The chromatographic analysis shows that spilanthol (affinin) was the primary compound found in the EHFAo. Hence,* in silico* assessment was performed to evaluate the pharmacokinetic and toxicological properties of this molecule and 37 metabolites derived from it. Overall, our data show that the treatment caused no detrimental changes in progenitors regarding their gonads or fertility but caused some potentially teratogenic activity in embryos, which may be due to the action of spilanthol's metabolites M3, M6, M7, M8, M16, M28, and M31.

## 1. Introduction

The plant species* Acmella oleracea* (L) R. K. Jansen is popularly known as “jambú”, albeit other vernacular names are used, such as “agrião-do-Pará”, “agrião-bravo”, “botão-de-ouro”, “jambuaçu”, “abecedária”, “agrião-do-Brasil”, “mastruço”, “agrião-do-norte” [1], “jaguaçú”, “erva-maluca”, and “jagurama” [2]. This species, from the family Asteraceae, is native from oriental Amazon and is highly cultivated in the states of Pará and Amapá due to its relevance in local cuisine and folk medicine [3, 4].

The most representative compounds of this species are alkylamides, specially spilanthol ((2E,6Z,8E)-N-Isobutyl-2,6,8-decatrienamide), also called Affinin; this molecule is known for its pharmacological properties [5–7]. In folk medicine, inflorescences and leaves from* A. oleracea* are used to treat mouth and throat ailments, tuberculosis [2, 7, 8], as a diuretic agent [4, 9, 10], to treat flu and cough, as an antibacterial, antifungal, antimalarial [11–13], and insecticide [11, 14], and to treat rheumatism, as an anti-inflammatory, analgesic, and local anesthetic agent. The inflorescences are also used as a local anesthetic for toothaches [3].

Worldwide, researches are performed continuously to improve our understanding of how diseases work and how we can treat them. In this context, zebrafish (*Danio rerio*) is a remarkable model organism, particularly for assessment of acute [15] and reproductive [16–18] toxicity of compounds, either natural [19, 20] or synthetic [21].

Among the methods used to assess a compound's reproductive toxicity with zebrafish, the immersion method is highlighted due to its simplicity and reproducibility. The fish is exposed to a tested compound added to water, allowing the researcher to evaluate if this compound interferes in reproductive parameters, if it causes any toxicity-induced histological changes in the gonads, and finally if it causes any teratogenic or even lethal effects in the fish.

The metabolism of an initial drug or xenobiotic can produce metabolites with considerably different pharmacological and physical-chemical properties; this consequently has relevant implications regarding the safety and effectiveness of the compound [22, 23]. To reduce potential risks which caused the metabolism of a given drug, it is necessary to employ reliable methods to predict it. However, due to restrictions to study human metabolism of xenobiotic compounds, a computational approach is currently the method of choice [22].

Considering these information, this study aimed to evaluate the reproductive performance of zebrafish treated with the hydroethanolic extract from the flowers of* Acmella oleracea* (L) R. K. Jansen; evaluate the effects of the treatment on embryonic development of the offspring (F1 generation) from exposed progenitors; and perform* in silico* assessment of the principal molecule of EHFAo—spilanthol—and its metabolites, to appraise their potential toxicity to the human body.

## 2. Materials and Methods

### 2.1. Plant Material

*Acmella oleracea* (L.) R. K. Jansen flower samples were collected in September 2016, District of Fazendinha (S 0° 02′30.40 ^″^/W 5106′37.5 ^″^), in the City of Macapá, State of Amapá, Brazil. A dried plant specimen was stored in IAN Herbarium (Embrapa Amazônia Oriental, Bélem, Pará, Brazil), under the identification n° 196011.

### 2.2. *A. Oleracea* Flowers Hydroethanolic Extract (EHFAo) Preparing

Selected fresh flowers were ground to powder and then cold macerated for seven days in 70% hydroethanolic solution. The resulting solution was filtered and concentrated in a rotary evaporator (Quimis Model Q 218.2) at 40°C for complete evaporation of the solvent. Subsequently, this concentrate was freeze-dried, yielding 2.5%.

### 2.3. EHFAo Analysis: Ultra High-Performance Liquid Chromatography (UHPLC-ESI-MS)

EHFAo analysis was performed using an Agilent 1290 (Agilent®) Liquid Chromatograph with DAD detector, coupled to an Agilent G400 Triple Quadrupole Electrospray Mass Spectrometer in positive ionization mode.

Samples containing 5 mg/mL of the extract were prepared with methanol, filtered in microfilters, and then analyzed on a reverse phase column (ZORBAX XDB C8; 2.1 x 50 mm 3.5 micron), eluted with water and (A) 0.1% acetic acid and (B) acetonitrile (40:60) in isocratic mode, with 2 *μ*L of injection volume, flow rate of 0.05 mL/min, and 1,200 bars of pressure limit, in 13 min. of analysis time. The column temperature was kept at 40°C, the thermostat at 20°C and the samples were kept at room temperature. The compounds were detected at 230 nm. Mass spectrometry was performed through electrospray ionization in full scan mode, operating between 50 and 700 m/z, with 50 V of collision energy. Nitrogen gas was used as nebulizer (45 psi), with a flow rate of 5 L/min in positive mode. The mass found was registered in positive ionization mode, and the spectra of the fragments were identified according to the literature.

### 2.4. Animals

The project of this research was approved by the Animals Use Ethics Committee (CEUA) from the Federal University of Amapá (UNIFAP), under registration n° 002/2018.

Both sexes of* Danio rerio* (n = 48 fishes) were used (AB wild-type). The animals were about six months old, 3.5-4.0 cm in length and 650 mg in weighing. All animals were bought from the company Acqua New Aquarium and Fish Ltda. (Igarassu, PE, Brazil), kept in quarantine, and then acclimated over a month before the experiments, performed in the Zebrafish Room from the Drugs Research Laboratory of the Federal University of Amapá (UNIFAP).

The fish were kept in water tanks (25 L) equipped with running water system at temperatures between 25-29°C; pH between 8,4 and 8.6; hardness between 140 and 145 mg/l CaCO_3_; and 90% of dissolved O_2_. The fish were stored in the proportion of 1 fish per water liter, in a light/dark cycle of 12/12 hours. All animals were fed twice a day with ration flakes (Alcon Colours) [15].

### 2.5. Experimental Design

After the acclimation period, the animals (n = 48) were randomly divided into four groups (n = 12/group), with a proportion of two male fishes for each female fish.

  The groups were divided as follows:
  A: parental generation treated with regular water from the system (control group);
  B: parental generation treated with EHFAo at 50 *μ*g/l diluted in 1 L of regular water;
  C: parental generation treated with EHFAo at 100 *μ*g/l diluted in 1 L of regular water;
  D: parental generation treated with EHFAo at 200 *μ*g/l diluted in 1 L of regular water.

One week before the experiments, a pretreatment was performed with all groups using only regular water from the system. During this period, the eggs were collected, quantified, and evaluated until 96 hpf. This procedure was carried out to establish base values for each group.

After this pretreatment, the groups were treated according described throughout 21 days. Every day, 50% of water from the tanks was renewed, containing the same original concentration of EHFAo designed for the respective group, prepared from a mother solution tank, changed weekly [16]. The concentrations used over the experiment were established after a preliminary acute toxicity test (48 h), in which eggs, mortality, and teratogenesis were evaluated until 96 hpf.

### 2.6. Egg Collection and Maintenance

After mating and spawning, fertilization occurred about 30 minutes after the lights were turned on. The eggs were collected, quantified, and washed using regular water from the system and then stored on inert Petri dishes with regular water (changed 70% daily); these Petri dishes were stored in a stove (SOLAB SL-102/630) at 28°C ± 2 until 96 hpf [24].

### 2.7. Teratogenesis and Lethality Evaluation

A daily observation was performed to evaluate the embryos in 24, 48, 72, and 96 hpf. The embryos were classified according to the severity of morphological defects and signs of toxicity (Table 1).

### 2.8. Gonads Histopathological Analysis

After the 21 days of exposure to EHFAo, the animals were euthanized according to the method described by Castro [25]. After this procedure, the specimens were fixed in Bouin solution, decalcified using EDTA at 7%, processed and stained with Hematoxylin & Eosin, as described by Souza et al. [15]. Histological changes were qualitatively and quantitatively appraised as described by Souza et al. [15].

### 2.9. Statistical Analysis

The values of eggs deposition were expressed as a mean ± standard error of the mean (SEM). To evaluate morphologic deviations among embryos compared to the control group was performed the bidirectional analysis of variance Kruskal-Wallis, followed by the post hoc test of Dunn. Results with p < 0.05 were considered statistically significant. All statistical analysis was performed using the software GraphPad Prism v. 5.0.

### 2.10. *In Silico* Evaluation of Spilanthol Metabolism

Spilanthol and its metabolites were appraised using the online server PreADMET (https://preadmet.bmdrc.kr/). This server calculated pharmacokinetic properties, such as solubility in pure water and skin permeability (P_Skin_). PreADMET predicts* in vitro* values of P_Skin_, and the result is given as log Kp.

**Kp** (cm/hour) is defined, according to Singh [26], as

(1) $$\begin{array}{c} Kp=Km*\frac{D}{h} \end{array}$$

**Km** represents the coefficient of distribution between the stratum corneum and vehicle;** D** represents the average diffusion coefficient (cm^2^/h); and** h** represents the thickness of the skin (cm).

PreaADMET also predicted values of plasmatic protein binding (PPB), blood-brain barrier penetration (BBB), permeability in Caco-2 cells, toxicological properties—including environmental (using the species Minnow as a parameter) and developmental (using Medaka fish as a parameter) [27]—and the risk of cardiac toxicity due to inhibition of the human gene ether-a-go-go-related (hERG). The structures assessed were further analyzed using the software Derek [28], to compare its results with those of PreADMET.

## 3. Results

**Figure 1:**
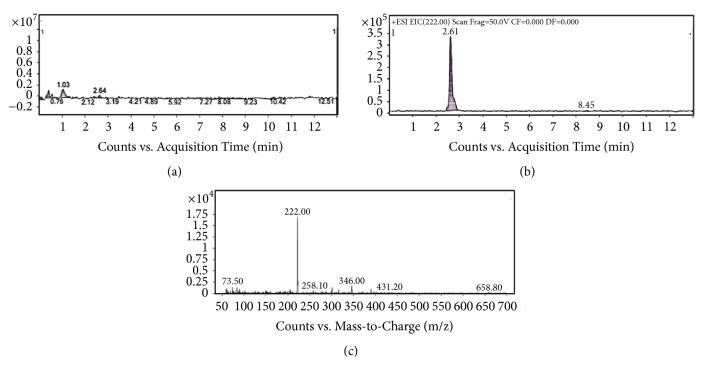
UHPLC-DAD-ESI-MS Analysis of the EHFAo: total ions chromatogram (a); extracted ions chromatogram (b); and mass spectrum in scan mode (c).

When analyzed through UHPLC-ESI-MS, the extract had a peak at 2.61 minutes corresponding to spilanthol in the chromatogram of total ions (TIC), with 22.56% of relative abundance, determined by integration of the peaks (Figure 1).

### 3.1. Gonads Histopathology

**Figure 2:**
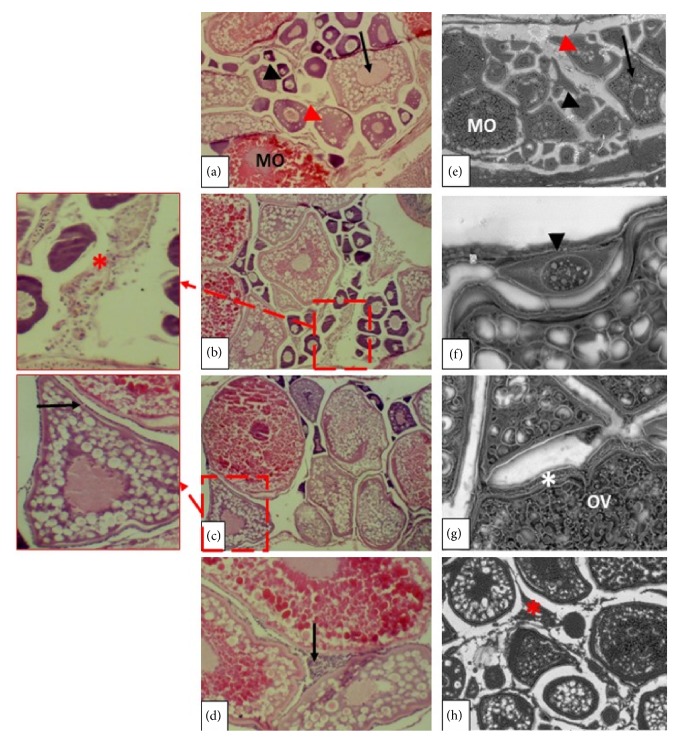
Longitudinal sections of zebrafish ovaries. In figures (a) (H&E), (e), (f), and (g) (SEM) normal aspects of zebrafish ovaries are observed, with perinucleolar oocyte (black arrowhead), primary vitellogenic oocyte (red arrowhead), vitellogenic oocyte (black arrow), mature oocyte (MO), chorion (asterisk), and vitellogenic oocyte (OV). In (b) (H&E) and (h) (SEM) interstitial fibrosis is observed (red asterisk), present in all groups treated with EHFAo. In (c) oocyte atresia is observed (black arrow), observed only in the group treated with EHFAo at 200 *μ*g/L. In figure (d) hyperplasia of perifollicular cells is observed (black arrow), observed in all treated groups.

In females from the control group, all follicles—such as the perinucleolar, alveolar cortical, vitellogenic, and mature follicles—were regular (Figures 2(a) and 2(e)).

After 21 days of treatment with EHFAo (at 50, 100, and 200 *μ*g/L), mild alterations were observed in females' ovaries from the treated groups, including perifollicular cells hyperplasia (a level II severity change found in < 20% of the tissue), as shown in Figure 2(d). Moderate alterations were also observed, like interstitial fibrosis (level III severity change in ≤ 30% of this tissue), as shown in Figures 2(b) and 2(h). Finally, oocytes atresia was observed, but only in females treated with the highest concentration (200 *μ*g/L), as shown in Figure 2(c).

**Figure 3:**
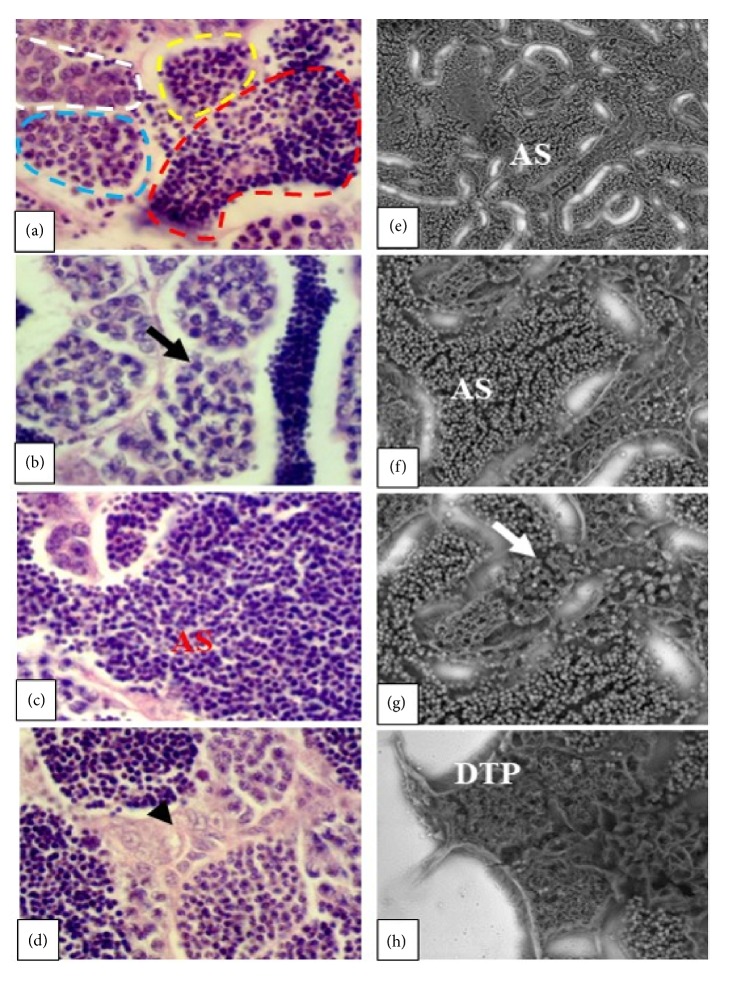
Longitudinal sections of zebrafish testes. In figures (a) (H&E), (e), and (f) (SEM) normal aspects of zebrafish testes are observed, with mature spermatozoa (red dashed lines), spermatocytes (blue dashed lines), type-II spermatocytes (white dashed lines), and plenty of spermatozoa (AS). In figure (b) (H&E) and (g) (SEM) the development of asynchronous gonads is observed (black and white arrow) in groups treated with EHFAo at 100 and 200 *μ*g/L. In figure (c) (H&E) plenty of mature spermatozoa is observed (groups treated with EHFAo at 100 and 200 *μ*g/L). In (d) (H&E) hyperplasia of interstitial cells is observed (arrowhead), in the group treated with EHFAo at 50 *μ*g/L. In (h) (SEM), testicular parenchyma degeneration is observed (DTP), observed in groups treated with EHFAo at 100 and 200 *μ*g/L.

In males' testis from the control group, it is observed that the parenchyma is organized with numerous highly developed spermatocytes, all cell components discernible, and abundant normally distributed spermatozoa. In the interstice several conjunctive cells and blood vessels are seen. Leydig cells are regularly arranged, with round nuclei, sometimes forming agglomerates in the interstitial space. Lastly, the spermatozoa had round-shaped heads, although some were oval-shaped (Figure 3(b)).

In males exposed to EHFAo at 50 *μ*g/L no sign of severe histopathological changes was observed; only interstitial cells had barely discernible hyperplasia (< 20%; a level I severity alteration), as shown in Figure 3(d).

Among male animals treated with EHFAo at 100 and 200 *μ*g/L, few cellular features were altered, for instance, clear signals of testis parenchyma degeneration were detected, a level III severity alteration (Figure 3(h)). In animals exposed to EHFAo at 100 *μ*g/L, this alteration was present in 50% of the tissue, while those exposed to EHFAo at 200 *μ*g/L had 60% of the tissue altered. In these groups the highest percentage of mature spermatozoa (Figures 3(c), 3(e), and 3(f)), hyperplasia of interstitial cells, and development of asynchronous gonads was observed, a level II severity alteration, in ≥ 20% of the tissue (Figures 3(b) and 3(g)).

### 3.2. Fertility

**Figure 4:**
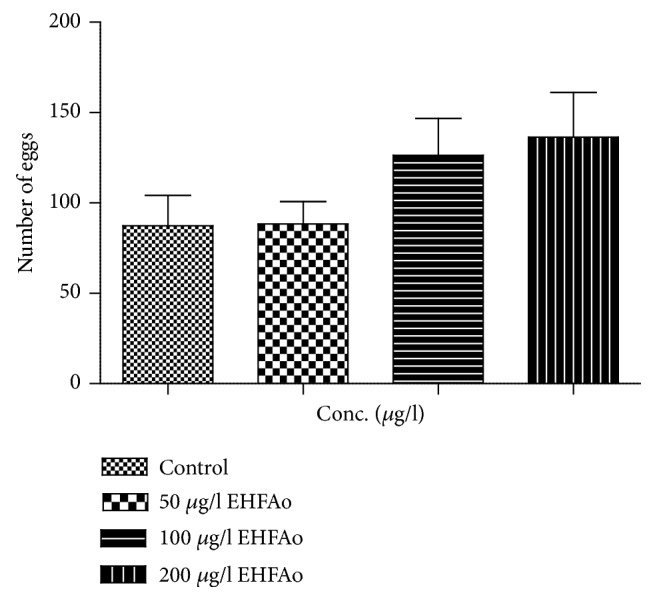
Average values of eggs production in zebrafish over the treatment period (21 days) with EHFAo. The eggs deposition was higher in groups treated with the two highest doses of EHFAo. The numbers of eggs from each group were compared among each other using the software GraphPad Prism 5.0, but the difference was not statistically significant (p > 0.05).

Figure 4 shows the average number of collected eggs over 21 days of treatment with EHFAo. In the period before treatment, the spawning occurred in regular intervals among all groups, with 100% of fertilization and hatching.

The highest numbers of deposited eggs were observed in the groups exposed to EHFAo at 100 and 200 *μ*g/L (126.5 ± 20.2 and 136.4 ± 24.7, respectively); a total of 18 spawns were registered in the group treated with the concentration 100 *μ*g/L and 15 spawns in the group treated with the concentration 200 *μ*g/L. The treatment resulted in an apparent concentration-dependent increase of spawning.

### 3.3. Embryos Lethality and Teratogenesis Assessment

**Figure 5:**
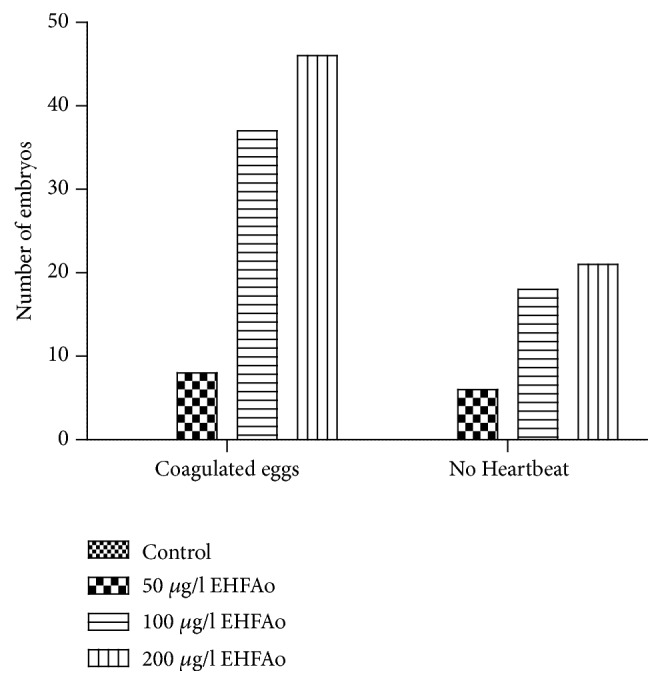
The graph shows the numbers of embryos with lethal abnormalities. The groups treated with EHFAo at 100 and 200 *μ*g/L had the highest incidence of lethalities, and coagulation was the most frequent egg abnormality.

Lethal effects observed in the embryos, whose progenitors were exposed to EHFAo, included coagulation and absence of heartbeats, registered up to 96 hpf. These lethalities were more prominent in the groups whose progenitors were exposed to the highest concentrations (Figure 5). Mortalities caused by the occurrence of several alterations in one single embryo were also registered up to 96 hpf.

**Figure 6:**
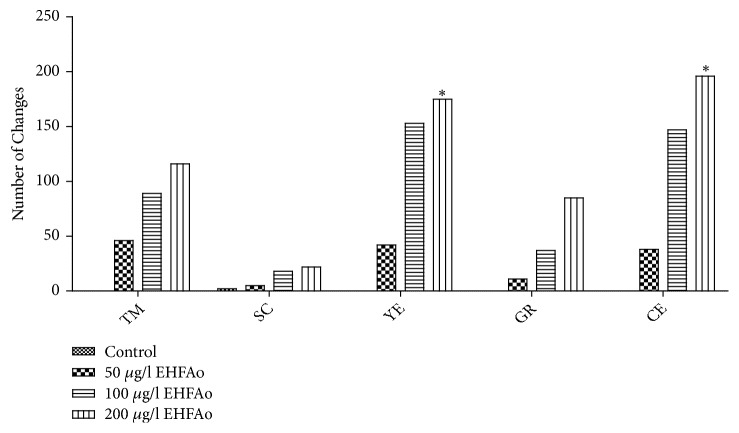
Embryonic and larvae changes in the offspring from the parents exposed to treatment with EHFAo for 21 days. *∗* p < 0.05, statistically significant difference compared to the control group; TM: tail malformation, SC: scoliosis, YE: yolk edema, GR: growth retardation, and CE: cardiac edema.

The number of embryos with at least one malformation was higher between 48 and 72 hpf. In this period, all larvae had already hatched (Figure 6), allowing a better view of embryos' structure. After 96 hpf, occurrences of malformations decreased in all groups.

**Figure 7:**
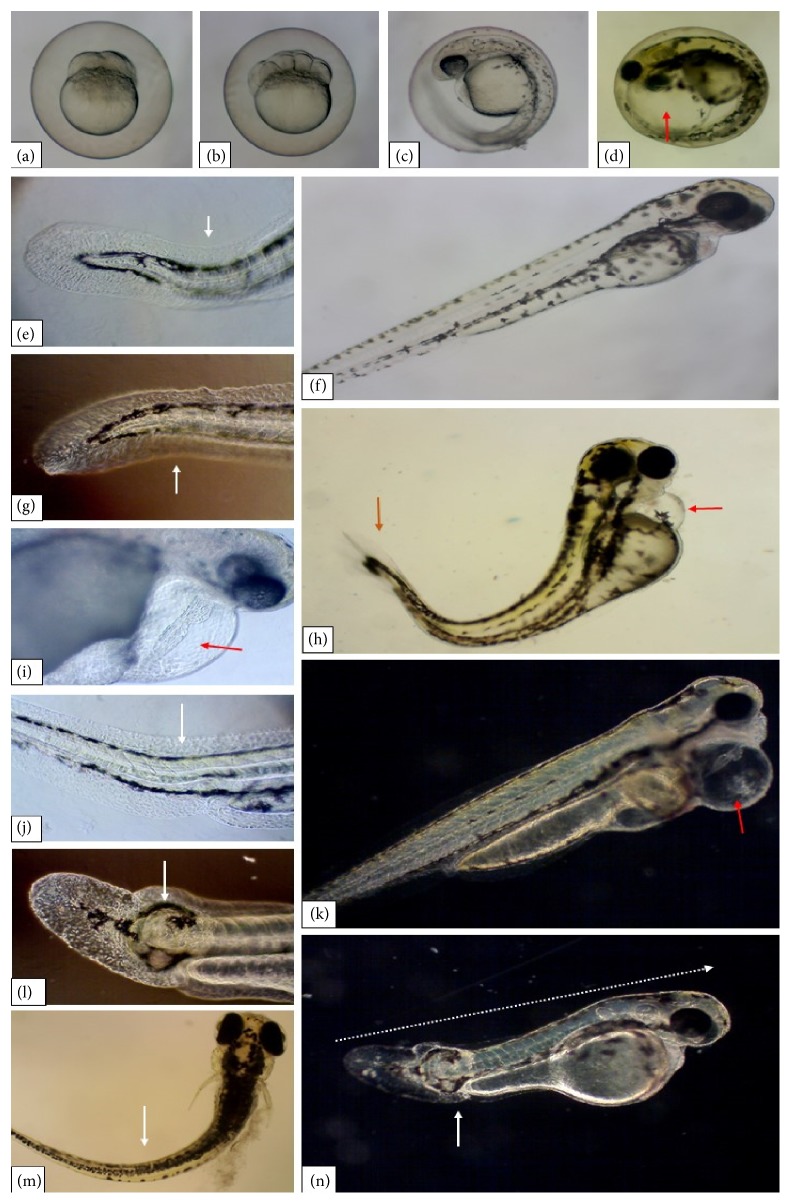
Representative pictures of zebrafish embryos and larvae from groups (a), (b), (c), and (d), whose parental generation was exposed to EHFAo at 50, 100, and 200 *μ*g/L. (a) Normal embryo with two cells (0.75 hpf); (b) normal embryo with 16 cells (1.5 hpf); normal embryo (c); and larva (f) at 24 and 48 hpf. White arrows indicate tail malformation in (e), (g), (h), (l), (m), and (n). Cardiac edema in 24 hpf embryo is shown in (d), and 96 hpf larvae in (i), (h), and (k) (red arrows). In (n) a fish with retarded growth is observed (white dashed arrow).

The most severe and evident malformations recorded were yolk and cardiac edema (Figures 7(d), 7(h), 7(i), and 7(k)). The total number of cardiac edema occurrences was 38, 147, and 196, in groups B, C, and D, respectively, while the number of yolk edema occurrences was 42, 153, and 175. However, only group D—whose progenitors were exposed to EHFAo at 200 *μ*g/L—had a statistically significant difference compared to the control group (A). Larvae with such malformations did not survive after 96 hpf.

Tail deformity (Figures 7(e), 7(h), 7(j), 7(l), 7(m), and 7(n)) and retarded growth (Figure 7(n)) were frequently observed alterations, but without a statistically significant difference when compared to the control group. These alterations resulted in impaired larval movement, which was evident in groups C and D. The least frequent alteration was scoliosis, with two occurrences in group A, 5 in B, 18 in C, and 22 in D.

### 3.4. Spilanthol Metabolism* In Silico* Assessment

**Figure 8:**
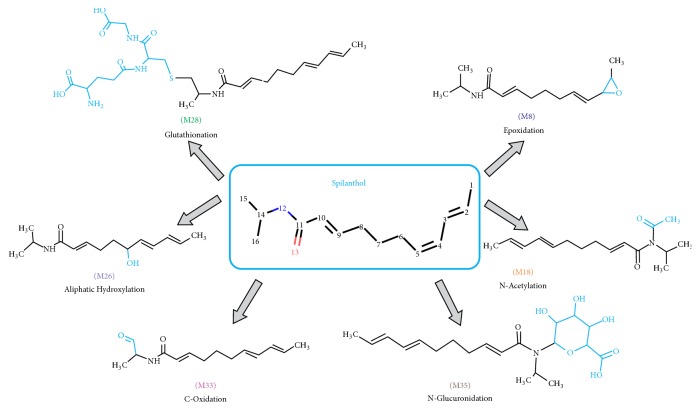
MetaTox results from spilanthol and its metabolites with their respective chemical reactions.

Figure 8 shows spilanthol metabolites predicted by the software MetaTox and the reactions to form them. The software DL_50_ value for spilanthol was 20.80 mg/kg in rats. Among the chemical reactions predicted by the enzyme CYP450 (Figure 8) were aliphatic hydroxylation, C-oxidation, N-glucuronidation, N-acetylation, epoxidation, and glutathionylation.

As shown in Table 2, the metabolite M8 is formed through epoxidation and catalyzed by epoxide hydrolase. According to the software, M8 has a potential risk to cause cardiac insufficiency and is potentially carcinogenic for male rats and female mice. M33 is a phase I reaction (oxidation) metabolite and was predicted to be potentially carcinogenic in the liver of male mice, in the lungs of female mice, and for tumor bearer male rats [29]. The metabolite M18 was predicted to be formed through N-acetylation, one of the major routes of biotransformation from xenobiotics with an aromatic amine (R-NH_2_), which are converted into aromatic amides (R-NH-COCH3) [30].

**Figure 9:**
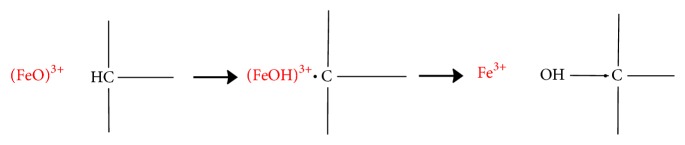
P450 enzymes catalyze hydroxylation preferably. Adapted from [23].

The metabolite M26 is formed through aliphatic hydroxylation, as shown in Figure 9. In simple hydrocarbons with linear chain, aliphatic hydroxylation occurs in terminal methyl groups and internal methylene groups. The oxidation of some aliphatic alkenes produces metabolites reactive enough to bind covalently to the heme portion of cytochrome P450. M26 showed no side effect.

As for M28 and M35, both are phase II metabolites. M28 is formed through a conjugation reaction with the tripeptide glutathione (Gly-Cys-Glu). The products of conjugation between glutathione and xenobiotics are fundamentally different from those formed by conjugation with other amino acids and dipeptides [31]; M28 showed no side effect but was predicted to be potentially carcinogenic for the kidney of male mice and skin of male rats. The metabolite M35 is formed through N-glucuronidation; this reaction requires the cofactor uridine diphosphate glucuronic acid (UDPGA) and is catalyzed by the enzyme UDP-glucuronosyltransferase (UGTs), which is located in the endoplasmic reticulum of the liver, and other tissues, such as the kidney [32]. The site of glucuronidation is often an electron-rich nucleophilic heteroatom, which in the case of spilanthol is the nitrogen. As side effects, M35 showed to be toxic to nephrons and carcinogenic to the kidney of male mice.

### 3.5. In Silico Assessment of Spilanthol Pharmacokinetics

The prediction of Absorption, Distribution, Metabolism, Excretion, and Toxicity (ADMET) for spilanthol and 37 metabolites formed from it is shown in Table 3; this table also shows values of solubility in pure water, absorption (P_Skin_ and Caco-2 cells), among others.

The solubility in pure water is a property of singular interest in drug development since it is directly related to the compound's pharmacokinetics. Spilanthol had a value of 384.737 cm/h, as shown in Table 3. In this study, the highest P_Skin_ values were those of spilanthol (-0.860042), M9 (-0.992692), M21 (-0.996746), and M22 (-0.994307). Most values of permeability in Caco-2 cells (P_CaCo2_), shown in Table 3, are comprised between 4 and 70; the minimum value of this property was for M34 (10.5575 nm/s), and the maximum values were for M8 (56.2978 nm/s) and spilanthol (53.0118 nm/s). None of the molecules had a high permeability value (≥ 70). The metabolites M13, 15, 16, 21, 25, 30, 36, and 37 had values inferior to 4.

Moreover, distribution properties (PPB % and C_Brain_C_Blood_) were evaluated for spilanthol and its 37 metabolites. Spilanthol and the molecules M1, M3, M4, M6, M9, and M21 had a plasma protein binding index (PPB) equal to 100%.

From 38 molecules assessed (Table 3), 20 of them were classified as active in the central nervous system, with C_Brain_C_Blood_ values > 1. From these, the highest values were from spilanthol (6.711), M1 (2.065), M2 (2.491), M3 (2.662), M4 (2.771), M5 (2.572), M6 (2.669), M18 (2.245), and M21 (2.245). Molecules with C_Brain_C_Blood_ values < 1, on the other hand, are classified as inactive in SNC; among these molecules are M12, M13, M23, M24, M27, M29, M35, M36, and M37, whose values ranged between 0.030 and 0.038.

As shown in Table 3, M10 and M21 have a high probability of being hERG-type K^+^ channels blocker; 13 other metabolites were classified as ambiguous, and 23 had a medium probability of causing this effect. Also, these metabolites showed no probability to be toxic environmentally (Minnow) or in the development (Medaka).

Additional toxicological data for spilanthol and its 37 metabolites tested using the software Derek are shown in Table 4. Overall, this data indicates that spilanthol and 18 of its metabolites show toxicity to skin sensibility. According to the software Derek, this property is due to the pharmacophore groups: alpha or beta-unsaturated amide or precursor, alpha or beta-unsaturated ketone or precursor, epoxide, aldehyde, and alpha or beta-unsaturated aldehyde or precursor. The molecules M3, M6, M7, M8, M16, M28, and M31 had a probability of causing chromosome damage by inducing molecular structural changes. According to the software Derek, this potential to cause chromosome damage is due to the presence of alpha and beta-unsaturated ketones (in M3, M6, M28, and M31) and epoxide (in M7, M8, and M16).

As shown in Table 4, nine molecules were attributed of being potentially carcinogenic to humans due to the pharmacophore groups: alpha or beta-unsaturated aldehyde, ketone or imine; epoxide; and alpha or beta-unsaturated amide, nitrile, or nitro compound. Other molecules were attributed to being potentially toxic in the development (M7, M8, and M16), cause eye and skin irritation in humans (M7, M8, M16, and M33) or irritation in the respiratory tract (M33). From the 38 molecules screened, 23 had none toxicologic alert for humans.

## 4. Discussion

In mass spectra analysis of the EHFAo (Figure 1), there is a peak with retention time equal to 2.64 minutes and 22.76% of relative area; this peak corresponds to the fragment m/z = 222 (base peak), which according to the literature is spilanthol ((2E, 6Z, 8E)-N-isobutyldeca-2,6,8-trienamide). Spilanthol is an alkylamide, which are molecules with a medium to long fatty acid chain (8 to 18 carbons), often aliphatic and with one amide group [33]. The extracted ions chromatogram was analyzed to find out the exact retention time of peak from spilanthol, and it was observed that the peak base m/z = 222 was eluted in 2.61 minutes.

Some other fragments were detected in product ions analysis, the fragment m/z 69 can be attributed to the group isobutylnitrile (C_4_H_7_N^+^), while the fragment m/z 53 can be C_3_H_3_N^+^, and m/z 41 can be C_2_H_3_N^+^. The fragment m/z 29 suggests a loss of carbonyl group from spilanthol, and the fragment m/z 149 indicates rupture of a C-N bond, with loss of the amine group. Singh and Chattuverdi [34] also detected spilanthol in the leaves of* Spilanthes acmella* through electrospray ionization, evidenced by a base peak m/z = 222. These authors also reported a fragment m/z = 99, which could be isobutyl isocyanate (C_5_H_9_NO).

Spilanthol has several pharmacological activities reported [35], among them, its local anesthetic activity is the most reported [7, 35–42]; other activities are analgesic [43], antioxidant [39], anti-inflammatory [38, 44, 45], antiwrinkle [46], antifungal [35], aphrodisiac [35], antimalarial [47], among others.

Until now, no research had been performed testing the hydroethanolic extract of* Acmella oleracea*, or its primary compound—spilanthol—over reproductive parameters of zebrafish or any teleost, neither its effect in the embryonic development of any model. In rats [48], EHFAo was reported to cause no maternal toxicity and significantly increased females' proestrus phase (88.91 and 444.57 mg/kg) when compared to a control group.

In this study, histopathology of female zebrafish ovaries showed oocyte atresia in the group treated with EHFAo at the highest concentration. This tissue alteration had also been reported to be caused by treatment with plant sterols, for instance, in studies with* Oryzias latipes* exposed to the phytoestrogens genistein, equol, and the bioflavonoid quercetin [49, 50]. Oocyte atresia is a natural process induced through apoptosis for ovarian maintenance; it is involved in growth and postovulatory regression of teleost fishes [51]. However, the increased occurrence of oocyte atresia can be a general response to endocrine disturbance and chemical-induced toxicity [52].

Hyperplasia and hypertrophy of perifollicular cells, observed in the groups exposed to EHFAo, are the increase of total number (hyperplasia) or size (hypertrophy) found in epithelial cells from granulosa or theca folliculi of a developing follicle [53]; this can be perceived as an increase of height and in the total number of granulosa cells, giving a pseudostratified layer appearance. Since perifollicular cells are involved in the production of aromatase in animals [54, 55], it is plausible that the increased size and number of these cells are a compensatory mechanism to maintain aromatase in appropriate levels for vitellogenesis [53].

Interstitial fibrosis occurs with an increased amount of fibrous connective tissue inside testicular or ovarian interstice [53]; this alteration indicates chemical-induced chronic stress [53, 56]. In this study, all females exposed to EHFAo had this tissue alteration, sometimes accompanied by inflammation and accumulation of protein liquid.

The occurrence of protein liquid has been linked to treatments with estrogenic compounds, and the fluid is presumably vitellogenin or a derivative [53]. The increase of fibrous connective tissue and other interstitial tissue alterations are in accordance with others reports of exposure to endocrine disruptors (e.g., E2, EE2, nonylphenol, isoflavones, octylphenol, and PCBs) using either zebrafish [50] or other species (e.g.,* Oryzias latipes*,* Dicentrarchus labrax*,* Chalcalburnus tarichi*,* Cyprinodon variegatus*, and* Oreochromis niloticus*), both in testes and in ovaries [49, 57–63]. This data indicates that connective tissue proliferation occurs to replace damaged structures.

In male fishes, histopathology showed a high percentage of spermatocytes and mature spermatozoa in the groups treated with the two highest concentrations of EHFAo, indicating that exposure to the extract at these concentrations induced spermatogenesis; this was also reported in studies with fishes exposed to androgens and pulp mill effluent [64].

In the groups exposed to the two highest concentrations of EHFAo testicular parenchymal degeneration was observed; in this situation apoptotic germ cells are observed, characterized by cell shrinkage, nuclear condensation, and fragmentation. No inflammation was observed along with these cells. When testicular degeneration is too extensive, this may lead to local or general loss of germinal epithelium [53].

Development of asynchronous gonads was registered only in the groups exposed to the extract at 100 and 200 *μ*g/L. This term is used to denote spermatocytes containing a mix of spermatocytes and spermatids, or spermatocytes containing primary spermatocytes in more than one meiotic phase [53].

The effects of EHFAo in eggs production and fertilization seem to be due to its influence over gonads' steroids. In this study an increase of eggs production in the treated groups was observed when compared to the control group. The results suggest that exposure of fishes to EHFAo stimulated the production of sexual hormones, which could be through modification of steroid enzymes or an indirect feedback effect [65], resulting in increased production of egg cells and spermatozoa.

Other studies were performed to assess the effect of* Acmella oleracea* extract over the sexual behavior of male rats [66] and maternal toxicity in female Wistar rats [48]. These studies indicate a potential aphrodisiac activity in* A. oleracea*, attributed to its N-alkylamides. However, until the present study, no assessment had been performed over its effects in the embryonic development.

Even though the treatment upregulated reproductive parameters in parents, it negatively affected the embryonic development of the offspring, evidenced by an increased rate of mortality and developmental defects, such as cardiac and yolk edema, tail malformation, and scoliosis. Some other authors had reported these developmental defects caused by treatment with other compounds [67–69].

Lethal and detrimental effects observed were more pronounced with the two highest concentrations of EHFAo, showing that these concentrations can be harmful to the offspring, and this toxicity is passed somehow from the treated parents to them. It is important to notice that the control group had no increase of lethality rates, and only two larvae had scoliosis (72 hpf). Other compounds were reported to induce developmental toxicity in the offspring after parental treatment, such as bisphenol S [70], fusaric acid [71], selenomethionine [72], ZnO nanoparticles [73], and azoxystrobin [74].

Based on previous studies, some mechanisms proposed to explain a compounds' toxicity induced in the offspring after treatment of parental generation are as follows:

(1) The treatment could induce alterations on egg cells and spermatozoa, resulting in affected development of the offspring [75, 76].

(2) Bioaccumulation of the compound and its metabolites in parental fishes could further induce its deposition from the eggs to the larvae, even if these compounds are absent in water [77–79].

(3) The embryos could absorb aqueous chemical products from parental fish tanks after spawning [76].

However, since the increased rates of lethalities and detrimental effects were accompanied by increased eggs deposition, the former could be a consequence of the latter, since the parental resources to produce a regular number of eggs (e.g., yolk) would be distributed among a higher number, resulting in less resource per egg, and hence favoring detrimental effects. Still, it is not possible to fully elucidate the mechanism of which the EHFAo induces toxicity to the offspring; this is due to an impossibility to measure the transference of EHFAo's compounds or derived metabolites from the parent to the embryo.

Some of the pharmacokinetic properties of isolated spilanthol or extracts containing it were already assessed in vivo or in vitro; in both cases the* in silico* values reported here can be helpful. In the total absence of any data, these values can serve as a parameter for new studies; if there is data, the results can be compared. It is important to notice also that some studies were performed with solutions containing spilanthol, and the in silico values here are deducted from spilanthol alone.

The water solubility of a compound is useful due to its influence in pharmacokinetic properties. The* in silico* prediction of spilanthol solubility in pure water was 384.737 cm/h. With 221.34 g/mol, this molecule is lipophilic (LogP = 3.39) [80], and despite the low solubility in water of spilanthol alone, the whole extract (EHFAo) could be fully dispersed in the water tanks at the concentrations used. The probable physical form of spilanthol is micelles, like other N-alkylamides [81].

Skin permeability is a crucial parameter mainly for transdermal administration of drugs and to evaluate the risks of a chemical that could accidentally touch the skin [82]. In this study, spilanthol and its 37 compounds had negative values of skin permeability, indicating high permeability of these molecules. This is in accordance with Boonen et al. [5] who tested the permeability in human skin of spilanthol-containing solutions with different solvents, such as 65% ethanol and 10% propylene glycol using a Franz diffusion cell system; overall, these authors showed that the spilanthol-containing solutions were able to permeate the skin. Also, Spiegeleer et al. [81] showed that spilanthol could be used to enhance skin permeability through increased partitioning of the molecules.

Caco-2 cells are currently being employed as the primary cell model to assess a compound's permeability and absorption. Table 3 shows the values of permeability in Caco-2 cells from spilanthol and its metabolites, and most values are average (4 ≤ x ≤ 70; spilanthol: 53); this is in accordance with Veryser et al. [80], who showed in vitro that spilanthol could permeate Caco-2 cells from the apical to the basolateral side and vice versa, which this was further confirmed* in vivo* in rats intestinal lumen. According to these authors, the lipophilic nature of this molecule favors transcellular transport. Boonen et al. [83] also showed that the ethanol extract of* Spilanthes acmella* (=* Acmella oleracea*) could permeate buccal mucosa in a Franz diffusion cell system.

The binding between plasma protein-drug can affect this drug's half-life; also, the bound moiety can act as a chemical reservoir for the drug, since the bound drug will be released to maintain the balance, while the unbound moiety will be metabolized and excreted from the body [84, 85]. As shown in Table 3, spilanthol and the molecules M1, M3, M4, M6, M9, and M21 had the highest PPB values. No* in vitro* or* in vivo* studies with this property were found.

The blood-brain barrier penetration index (BBB), shown in Table 3, is another crucial aspect of a drug. As shown in Table 3, spilanthol BBB was > 1, indicating that this molecule can pass through BBB; this is in accordance with Veryser et al. [80], who showed that spilanthol could rapidly cross the BBB in mice due to its lipophilic nature.

The human ether-a-go-go-related gene (hERG) is codified for a protein that forms a voltage-dependent potassium ion channel found in heart and nervous system; this channel is essential for the repolarization during the cardiac action potential. Conductance alterations of this channel, especially channel block, can lead to an impaired action potential [86]. Due to its importance in the regulation of the cardiac action potential, drugs that can interact with hERG are currently being taken out of the market, since this can result in cardiac arrhythmia and sudden death [86, 87]. In this study, only the molecules M10 and M21 had a considerable risk to interact with hERG.

Minnow and Medaka indexes indicate environmental and developmental toxicity, respectively. Their values were not significative, which is in accordance with Farias et al. [87], who evidenced that natural molecules induce none or few of these toxicity values. Note that this lack of developmental toxicity* in silico* corroborates the hypothesis that the detrimental and lethal effects observed in zebrafish embryos are more likely to be due to the increased egg deposition than a direct toxic effect by the extract.

The occurrence of skin sensitization alert indicates that a molecule can potentially induce skin sensitization, but it also depends if this molecule is absorbed through the skin. Usually, small lipophilic molecules pass more easily through skin and hence are more likely to induce sensitization. As shown in Table 4, 18 molecules were predicted to induce sensitization; this alert is due to the presence of amides, ketones, and alpha or beta-unsaturated precursors that can interact with skin proteins through a Michael addition reaction [88].

In this study, seven molecules were predicted of being potentially injurious for chromosomes. This alert is due to the presence of alpha or beta-unsaturated ketones (vinylketones), which can induce chromosomal aberration* in vitro* and in the L5178Y TK +/- assay. These compounds manage to induce weak positive results in relatively cytotoxic concentrations, such as alpha-ionone [89] and phorone [90]. On the other hand, negative or ambiguous results were attributed to some alpha and beta-unsaturated ketones in the micronucleus test* in vivo*, like curcumin [91], 2-cyclohexene-1-one [92, 93], and methyl vinyl ketone [94, 95].

Alpha and beta-unsaturated ketones possess an electrophilic center (Michael's acceptor) and hence, were attributed to react with DNA bases [96]; for instance, 2-cyclohexene-1-one and methyl vinyl ketone were shown to form guanosine adducts* in vitro* [97]. It is possible that the formation of such adducts can contribute to the potential of these molecules to induce chromosomal damage; also, there is some evidence that reactive epoxide species can be at least partially involved in the mutagenic activity of this kind of compound through metabolic activation [98].

Table 4 shows alerts of the potential genotoxic carcinogenicity found in 9 molecules, among them aldehydes, ketones, alpha, and beta-unsaturated imines. In laboratory conditions, alpha and beta-unsaturated compounds undergo a Michael-type conjugated addition with nucleophile species [99]; the carcinogenicity found in aldehydes and alpha or beta-unsaturated ketones is presumably due to nucleophilic attacks in their double bonds by DNA bases.

Epoxides are strong alkylating agents; their pharmacophore was identified as active in human developmental toxicity predicted in the molecules M6, M7, and M15; this occurs due to the opening of the molecule's ring, forming a reactive ion that can alkylate the DNA, culminating in adverse effects for the developing fetus. Diepoxides are usually more reactive than monoepoxides; however, carbonyl and thiocarbonyl groups can mitigate this reactivity. Currently, there are no studies reporting teratogenesis in animals or humans caused by treatment with these chemicals [100].

Eye, skin, and respiratory tract irritation alerts were registered in four molecules. This irritation is attributed to epoxy groups and due to their lower molecular mass (MM < 200), tends to be more irritating. The occurrence of two epoxide groups instead of one results in higher irritancy; however, low-mass (< 300) epoxides with low water solubility are usually only slightly irritating for eyes and skin [101].

Except for corrosive and highly reactive compounds, the potential to induce eye and skin irritation of a chemical relies on its physicochemical properties. Skin penetration is higher in low-mass (< 500) and relatively lipophilic molecules (Log P (octanol/water) = 1-4), while ocular irritation is higher in water-soluble compounds that dissolve quickly in the eye's tear film, cornea, and conjunctiva [102].

The software MetaTox is the first web application in which the generation of a metabolic pathway is coordinated with acute toxicity prediction. This website is useful at the beginning of drug development and offers some clinical advantages and cost reduction [25]. Some reactions of spilanthol metabolism are linked to its toxicity, such as hydroxylation, C-oxidation, N-glucuronidation, N-acetylation, epoxidation, and glutathionation. Spilanthol and the metabolites M18, M26, M28, and M33 had no collateral effects predicted.

## 5. Conclusion

Currently, this is the first study evaluating the reproductive and developmental toxicity of the hydroethanolic extract of* Acmella oleracea* (EHFAo). In parent zebrafish, this treatment resulted in few gonads tissue alterations, without interfering in reproduction and significantly increased eggs deposition; this has important implications for studies of nonintentional pharmaceutical treatments. Moreover, the results show that parental treatment can only induce lethal and teratogenic effects in the highest concentrations of EHFAo (100 and 200 *μ*/L). However, the exact mechanism of which the extract could induce toxicity at these concentrations in the embryos could not be fully elucidated since it is not possible to measure its transference from mother fish to embryos. It is possible that detrimental effects could be due to the increased deposition of eggs since it would result in few resources per egg.

Considering prediction errors, the results of pharmacokinetics parameters are, in general, within the limits of clinical relevance given in the literature for water solubility, skin permeability, PPB, BBB, and hERG.

*In silico*, spilanthol and 18 of its metabolites had skin sensitization potential attributed to more than one pharmacophore groups, and the molecules M3, M6, M7, M8, M16, M28, and M31 were predicted to cause chromosomal damage. For spilanthol, metabolism reactions included aliphatic hydroxylation, C-oxidation, N-glucuronidation, N-acetylation, epoxidation, and CYP450 enzyme glutathionation.

## Figures and Tables

**Table 1:** Teratogenic and lethal effects observed in zebrafish embryos throughout the development period.

Developmental toxicity		24 hpf	48 hpf	72 hpf	96 hpf
Lethal effects	Coagulated eggs^a^	+	+	+	+
	No heartbeat^b^		+	+	+

Teratogenic effects	Tail malformation^c^		+	+	+
	Scoliosis			+	+
	Yolk edema		+	+	+
	Growth retardation^d^		+	+	+
	Cardiac edema		+	+	+

^a^ Coagulated eggs are milky white and look dark when seen under optical microscopy.

^b^ No record of heart beats for one minute.

^c^ Tail malformation occurred when an embryo had a curved, twisted, or hook-like tail.

^d^ Growth retardation was evaluated by comparing treated embryos with control ones (size, development stage). At 72 and 96 hpf, growth retardation was considered when embryo's size was less than 2.9 and 3.3 mm, respectively.

**Table 2:** Most probable metabolites for spilanthol, with their respective chemical reactions, probability values, and predicted DL_50_.

Metabolites	Phase type	Chemical Reaction	Probability (PR)	DL_50_
M8	Phase I reaction (Oxidation)	Epoxidation	0,9991	46.49 mg/kg
M18	Phase II biotransformation reaction	N- Acetylation	0,8985	26.28 mg/kg
M26	Phase I reaction (Oxidation)	aliphatic hydroxylation	0,8072	33.14 mg/kg
M28	Phase II biotransformation reaction	Glutathionation	0,7797	327.74 mg/kg
M33	Phase I reaction (Oxidation)	C-oxidation	0,6322	28.69 mg/kg
M35	Phase II biotransformation reaction	N-glucuronidation	0,5135	81.34 mg/kg

**Table 3:** Predictions of pharmacokinetic parameters of spilanthol and its metabolites.

Pharmacokinetic								
Metabolites	S_Pure water (cm/h)_	P_skin_	PPB_(%)_	BBB_(C.brain/C.blood)_	hERG	CaCo2	Acute Toxicity (LC_50_ – mg/l)	
							Medaka	Minnow
Spilathol	384.737	-0.860	100.00	6.711	m_risk	53.0118	0.0181	0.01775

M1	901.681	-1.163	100.00	2.065	m_risk	25.6981	0.0450	0.04509

M2	631.747	-1.167	93.224	2.491	m_risk	25.698	0.0386	0.0409

M3	310.115	-1.125	100.00	2.662	m_risk	25.6981	0.0353	0.03059

M4	310.115	-1.113	100.00	2.771	m_risk	25.6949	0.0359	0.03348

M5	631.747	-1.150	92.756	2.572	m_risk	41.9066	0.0553	0.03904

M6	310.115	-1.139	100.00	2.669	m_risk	25.6981	0.0359	0.03040

M7	1206.68	-1.728	96.916	1.336	m_risk	47.8535	0.0828	0.0801

M8	845.441	-1.683	83.732	1.576	m_risk	56.2978	0.0958	0.06987

M9	776.796	-0.992	100.00	1.106	m_risk	19.7788	0.0731	0.06476

M10	68578	-3.067	66.967	0.875	l_risk	20.9729	31.35	16.810

M11	3973.59	-1.185	87.469	1.954	m_risk	23.6658	0.0819	0.09516

M12	142.092	-2.934	31.644	0.037	ambiguous	0.611474	0.2308	0.3764

M13	252.203	-2.635	34.217	0.038	ambiguous	14.5677	0.2754	0.3786

M14	48.8697	-2.936	35.301	0.040	ambiguous	0.624655	0.1404	0.2630

M15	48.8697	-2.931	35.216	0.063	ambiguous	0.626143	0.1305	0.2620

M16	728.976	-1.608	83.202	1.444	m_risk	47.7725	0.0902	0.08946

M17	428.204	-1.099	90.967	0.429	m_risk	49.8748	0.0133	0.01459

M18	1366.64	-1.114	93.160	2.245	m_risk	23.6658	0.0643	0.07021

M19	3102.58	-1.190	89.623	1.804	m_risk	25.6981	0.0705	0.09478

M20	48.869	-2.910	28.384	0.043	ambiguous	0.645312	0.2711	0.2598

M21	1007.38	-0.996	100.00	2.958	l_risk	37.5329	0.0275	0.02863

M22	863.164	-0.994	88.489	1.786	m_risk	25.8079	0.0923	0.1140

M23	99.5542	-2.918	32.768	0.034	ambiguous	1.14185	0.2015	0.3296

M24	99.5542	-2.914	31.183	0.030	ambiguous	0.686328	0.291	0.3299

M25	3102.58	-1.195	61.667	1.811	m_risk	25.6981	0.1203	0.09362

M26	5553.90	-1.294	68.862	0.402	m_risk	26.3048	0.1695	0.1878

M27	86.740	-2.565	36.681	0.036	ambiguous	14.7041	0.2742	0.2633

M28	2344.76	-1.252	90.150	0.726	m_risk	34.1402	0.0555	0.08731

M29	118.492	-2.979	31.622	0.033	ambiguous	0.782091	0.5623	0.4349

M30	6320.38	-1.230	76.605	1.610	m_risk	18.0322	0.1370	0.1216

M31	2344.76	-1.258	62.141	0.727	m_risk	34.1402	0.135	0.08580

M32	699.357	-1.051	95.684	1.263	m_risk	27.5265	0.0472	0.0458

M33	2358.30	-1.161	90.422	1.133	m_risk	27.5255	0.0546	0.04960

M34	1569.72	-3.016	77.128	0.052	ambiguous	10.5575	0.1132	0.1838

M35	257.656	-2.846	33.003	0.033	ambiguous	0.625941	0.2805	0.3762

M36	257.656	-2.845	29.616	0.030	ambiguous	0.654714	0.1285	0.3777

M37	524.879	-2.830	31.206	0.038	ambiguous	0.561316	0.1639	0.4769

**Table 4:** Toxicity results according to the software Derek.

Metabolites	Toxicity Prediction Alert (Lhasa prediction)	Toxicophoric Group	Toxicity Alert
Spilanthol	Skin sensitization in human	alpha, beta-Unsaturated amide or precursor	Plausible

M1, M5, M9, M11, M18, M25, M26 e M30	Skin sensitization in human	alpha, beta-Unsaturated amide or precursor	Plausible

M3	Chromosome damage in vitro in human	alpha, beta-Unsaturated ketone	Equivocal
	Skin sensitization in human	alpha, beta-Unsaturated ketone or precursor alpha, beta-Unsaturated amide or precursor	Plausible

M6	Carcinogenicity in human	alpha, beta-Unsaturated aldehyde, ketone or imine	Plausible
	Chromosome damage in vitro in human	alpha, beta-Unsaturated ketone	Equivocal
	Skin sensitization in human	alpha, beta-Unsaturated amide or precursor	Plausible
	Skin sensitization in human	alpha, beta-Unsaturated ketone or precursor alpha, beta-Unsaturated amide or precursor	Plausible

M7, M8, and M16	Carcinogenicity in human	Epoxide	Plausible
	Chromosome damage in vitro in human		
	Chromosome damage in vivo in human		
	Developmental toxicity in human		
	Irritation (of the eye) in human		
	Irritation (of the skin) in human		
	Skin sensitization in human	Epoxide alpha, beta-Unsaturated amide or precursor	

M19	Carcinogenicity in human	alpha, beta-Unsaturated amide, nitrile or nitro compound	Equivocal
	Skin sensitization in human	alpha, beta-Unsaturated amide or precursor	Plausible

M28, M31	Carcinogenicity in human	alpha, beta-Unsaturated aldehyde, ketone or imine	Plausible
	Chromosome damage in vitro in human	alpha, beta-Unsaturated ketone	Equivocal
	Skin sensitization in human	alpha, beta-Unsaturated ketone or precursor alpha, beta-Unsaturated amide or precursor	Plausible

M32	Skin sensitization in human	Aldehyde alpha, beta-Unsaturated amide or precursor	Plausible

M33	Carcinogenicity in human	alpha, beta-Unsaturated aldehyde, ketone or imine	Plausible
	Irritation (of the eye) in human	alpha, beta-Unsaturated aldehyde	
	Irritation (of the respiratory tract) in human		
	Irritation (of the skin) in human		
	Skin sensitization in human	alpha, beta-Unsaturated aldehyde or precursor alpha, beta-Unsaturated amide or precursor	

M36	Carcinogenicity in human	alpha, beta-Unsaturated amide, nitrile or nitro compound	Equivocal

## Data Availability

The statistical, figures, and tables data used to support the findings of this study are included within the article.
